# The Experience of Parental Caregiving for Children With Medical Complexity

**DOI:** 10.1177/00099228221142102

**Published:** 2022-12-06

**Authors:** Jessica Teicher, Clara Moore, Kayla Esser, Natalie Weiser, Danielle Arje, Eyal Cohen, Julia Orkin

**Affiliations:** 1 Department of Paediatrics, University of Toronto, Toronto, ON, Canada; 2 Child Health Evaluative Sciences, Peter Gilgan Centre for Research and Learning, SickKids Research Institute, Toronto, ON, Canada; 3 Division of Paediatric Medicine, Department of Paediatrics, The Hospital for Sick Children, Toronto, ON, Canada; 4 Edwin S.H. Leong Centre for Healthy Children, Toronto, ON, Canada

**Keywords:** children with medical complexity, parenting, caregivers, quality of life, qualitative research

## Abstract

Children with medical complexity (CMC) have complex chronic conditions with significant functional impairment, contributing to high caregiving demand. This study seeks to explore impacts of parental caregiving for CMC. Fifteen caregivers of CMC followed at a tertiary care hospital participated in semi-structured interviews. Interviews were concurrently analyzed using a qualitative description framework until thematic saturation was reached. Codes were grouped by shared concepts to clarify emergent findings. Four affected domains of parental caregiver experience with associated subthemes (in parentheses) were identified: personal (identity, physical health, mental health), family (marriage, siblings, family quality of life), social (time limitations, isolating lived experience), and financial (employment, medical costs, accessibility costs). Despite substantial challenges, caregivers identified two core determinants of personal resilience: others’ support (hands-on, interpersonal, informational, material) and a positive outlook (self-efficacy, self-compassion, reframing expectations). Further research is needed to understand the unique needs and strengths of caregivers for this vulnerable population.

## Introduction

Children with medical complexity (CMC) represent a growing pediatric population.^1^ Children with medical complexity are defined by complex chronic conditions, functional impairment requiring medical technology support, high health care resource utilization, and significant caregiving demand.^1-4^ Parental caregivers shoulder a significant proportion of both the resource coordination and physical aid required to meet their CMC’s multiple health needs.^4^ As a result, caring for a CMC has been shown to affect families’ social and financial circumstances,^1,5,6^ including more than half of parental caregivers ending their employment to fulfill the caregiver role.^3,5^ Impact on caregiver physical and mental health has also been documented,^5,7,8^ with the dual role of parent and medical carer increasing mortality risk in parents of children with major congenital defects.^9,10^

There is a paucity of literature regarding the breadth of impact on parental caregivers for CMC.^1,4,11^ Understanding what contributes to and mitigates challenges faced by caregivers is an essential first step to developing tailored clinical interventions for this vulnerable population.^12^ Our study seeks to augment existing literature by exploring parents’ experience of caregiving for CMC. The term “caregiver” in this article refers to parental caregivers of CMC.

## Methods

### Study Design and Approach

This study employed a qualitative description methodology to investigate the lived experience of parental caregivers of CMC. Qualitative description has been identified as particularly appropriate for health disparity research in vulnerable populations and for topics relevant to health practitioners and policymakers. Through qualitative description, investigators endeavor to stay close to the data and present truths from the participants’ perspective.^13,14^

### Study Setting and Participants

This study was based at The Hospital for Sick Children in Toronto, Canada, a 450-bed tertiary care pediatric hospital with a Complex Care interdisciplinary program serving approximately 500 CMC from birth to age 18. The program helps families with care coordination, informed goal setting, and decision-making. To access the program, children must meet one or more criterion from each of the following: technology dependence and/or users of high intensity care (eg, medical ventilation, constant medical supervision), fragility (eg, severe or life-threatening conditions, conditions where intercurrent illness poses immediate serious health risk), chronicity (ie, condition is expected to last at least six months), and complexity (eg, involvement of at least five health care providers at three locations of care).^15,16^ Participants were excluded if their child was at end-of-life given unique caregiving demands and needs in this population, or if non-English speaking. Ethics approval was obtained from The Hospital for Sick Children’s Research Ethics Board.

### Data Collection and Analysis

Participants were recruited through contact by a known health care provider. Aligned with a purposive sampling approach for maximum variation,^17^ twenty-nine caregivers were identified by a clinical team member as possible study candidates, aiming to represent diversity in family structure, participant gender, social circumstance, immigration status, rurality, and services accessed; fifteen agreed to participate. Participants were interviewed by one of three study investigators following informed written consent. Baseline data were collected by demographic survey. A semi-structured interview guide was developed by the research team based on literature review and consultation with interdisciplinary clinical team members in The Hospital for Sick Children’s Complex Care Program, including pediatricians, nurse practitioners, and social workers.^18^ The guide was then iteratively refined according to emerging patterns in the concurrent data analysis. Questions in the interview guide were open-ended, designed to guide the participant to first share information about their family, current physical environment, personal health, professional development, interpersonal relationships, and family functioning, and then reflect on how their role as a caregiver for a CMC influenced each of their experiences. For instance, participants were asked: “Can you tell me about your employment/career, and your partner’s employment/career? How has your role as a caregiver to your child affected your career?” and “How would you describe your family’s ability to cope?” Interviews were audio-recorded and transcribed verbatim. Interviews ceased when theoretical saturation was achieved; ie, no new codes were found to occur in the data of the final coded transcript.^19^

Typical of the qualitative description methodology, data were analyzed using qualitative content analysis.^20^ All meaningful text units were identified and coded through line-by-line data analysis via NVivo 10 software.^21^ Codes were grouped by shared concepts and given a category name. Development of codes and categories occurred independently, simultaneously, and iteratively by three investigators with prior experience in qualitative methods and who received support and guidance from a researcher with expertise in qualitative analysis. Continuous comparative analysis confirmed that categories were derived from the data rather than imposed using an existing framework.^13^ A fourth investigator assisted in grouping categories into major categories to clarify emergent findings.

Several processes enhanced rigor in this study. Triangulation ensured quality of coding: authors had agreement on the initial coding, categories, and major categories. Credibility was enhanced by peer debriefing with Complex Care health care providers to confirm fit with clinical practice. Finally, investigators maintained a strong focus on authenticity, giving attention to participants’ voices and remaining true to the phenomena under study.^22^

## Results

Fifteen participants were interviewed, of whom two-thirds were mothers and one-third were fathers. Twelve of fifteen participants were married, and eleven participants had more than one child. Eleven participants worked outside the home part-time or full-time, and an equal number had a household income of $60 000 or greater. Additional demographic data are summarized in Tables 1 and 2.

**Table 1.** Demographics of Participants.

Demographics of participants (n = 15)	n
Relationship to child	
Mother	10
Father	5
Marital status	
Single or separated	3
Married	12
Number of children	
More than 1 child	11
Age of child with medical complexity, y	
0-2	2
3-6	7
7-9	3
10-11	3
Health status of child with medical complexity	
Met all criteria for medical complexity: technology dependence/user of high intensity care, fragility, chronicity, and complexity	15
Size of community	
Large or medium city	13
Small city or rural area	2
Housing status	
Owns home	11
Rents home	4
Education level	
Some trade, technical or vocational school, or business college	1
Diploma or certificate from community college, or nursing school	5
Bachelor or undergraduate degree, or teacher’s college	6
Master’s degree	3
Employment status	
Full time and paid	6
Part time and paid	5
Not employed and not looking for work	4
Family income (in Canadian dollars)	
10 000-19 999	2
40 000-59 999	2
60 000-79 999	6
80 000 or more	5
Income supports	
Welfare	5
Caregiving supports	
Homecare nursing services	10
Privately paid childcare services	5
Respite	5
Immigration status	
Born in Canada	10
Immigrated to Canada	5
Method of transport to hospital	
Driving	13
Driving plus additional modes of transport	2

**Table 2.** Additional Demographics of Participants’ Partners.

Additional demographics of participants’ partners (n = 12)	n
Education level of partner	
Some community college, or nursing school	1
Diploma or certificate from community college, or nursing school	3
Bachelor or undergraduate degree, or teacher’s college	5
Master’s degree	3
Employment status of partner	
Full time and paid	9
Part time and paid	1
Not employed and not looking for work	2

Qualitative content analysis revealed two central themes: affected domains of parental caregiver experience and core determinants of personal resilience.

### Affected Domains of Parental Caregiver Experience

**Figure 1.**
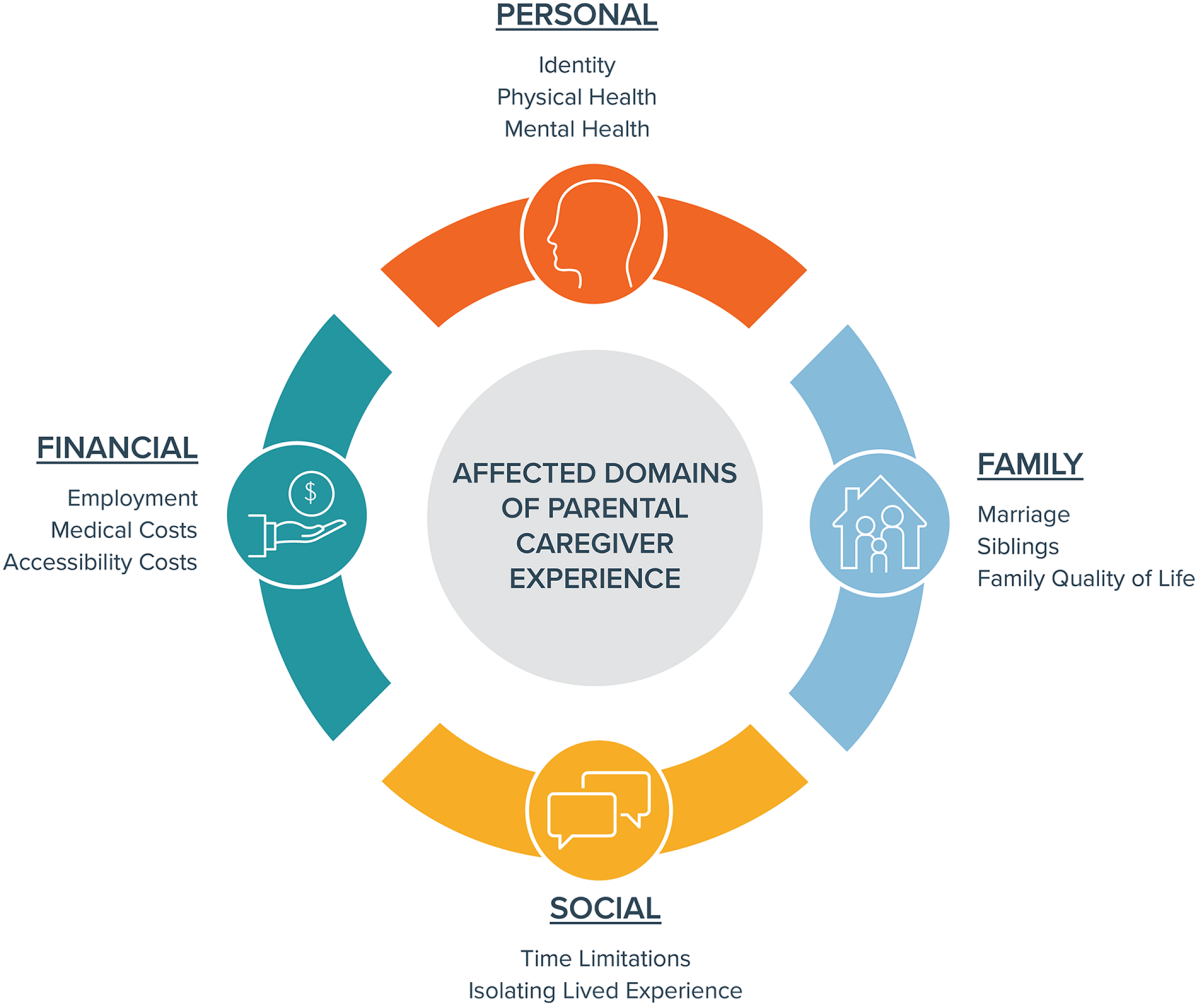
Affected domains of parental caregiver experience.

Four major categories were identified to describe affected domains of parental caregiver experience: personal, family, social, and financial. These are illustrated in Figure 1, with a selection of representative quotations in Table 3.

1. PERSONAL
  1.1. Identity: Several participants described that becoming a caregiver fundamentally changed every aspect of their lives. Some participants forewent established preferences, others rethought priorities or plans for the future. Participants expressed that their identity as ‘parent’ had been overtaken by roles including advocate, medical educator, and health care provider. A few caregivers found their new social and financial dependence on others incongruent to their identity of self-sufficiency.
  1.2. Physical Health: Many participants explained that the work of caregiving prevented them from attending to their own physical health. Examples included poor nutrition and weight gain, decreased exercise, sleep deprivation, musculoskeletal injuries from caregiving-related lifting, and inadequately accessing primary care.
  1.3. Mental Health: Some caregivers developed clinical depression or symptoms of profound sadness in response to their child’s diagnosis, prognosis, or perceived limitations; others reported emotions of anxiety or anger. Barriers to accessing mental health supports, including personal and systems-level availability were noted. Many participants raised themes of burnout adversely affecting coping.
2. FAMILY
  2.1. Marriage: Some caregivers cited their marriage as a critical source of support and collaboration in caregiving. Other marriages were strained by partners’ limited time together or inequity in caregiving responsibilities. Several participants described how health and financial stressors limited conversations with their spouse to discussion of their CMC’s needs. One participant separated from their spouse in part for caregiving respite through shared custody.
  2.2. Siblings: Caregivers with more than one child all raised concerns about limited time available to spend with siblings of the CMC. Most believed perceived prioritization of the CMC contributed to sibling jealousy, adversely affected behavior and emotional regulation, or strained the parent-sibling relationship. A few participants highlighted that being a CMC’s sibling promoted maturity in their other children.
  2.3. Family Quality of Life: Several participants described the negative impact on their family’s coping when the CMC is in poor health. Outside of acute illness, participants detailed how the enormous planning required for ‘simple’ activities involving the CMC meant resorting to activities with individual family members or limiting recreation options to ensure the CMC’s inclusion. One parent emphasized that caregiver respite involves separation from the CMC, inherently impacting family time. Several participants considered family quality of life in their housing decisions, eg, living near green spaces or in close proximity to medical care at the expense of other priorities.
3. SOCIAL
  3.1. Time Limitations: Participants described caregiving taking up the vast majority of their time, limiting or preventing social engagement with family, friends, or their religious community. One participant emphasized that their child’s strict medication and nutrition schedule presented a social limitation unique to caregivers of CMC.
  3.2. Isolating Lived Experience: Several participants discussed how their lived experience made it difficult to relate to anyone who was not also a caregiver for a CMC as others did not understand the complexity or extent of a CMC’s caregiving demands. Two participants labeled talking about their CMC with others as “the elephant in the room.”
4. FINANCIAL
  4.1. Employment: Caregiving limited participants’ employment either due to the child’s needs or to the mental health impacts of caregiving. Some participants described unemployment; others compromised salary or career advancement. Only some employers provided necessary accommodations including options to work remotely. Self-employed participants noted increased flexibility at the expense of stable income.
  4.2. Medical Costs: Participants cited significant medical costs associated with caregiving which were inadequately covered by social assistance, including hospital expenses, private childcare, medications, and rehabilitation programming; even some ‘covered’ costs were reported to require substantial upfront payment with subsequent reimbursement. For some caregivers, the greatest financial impact was on savings or debt management as day-to-day or unexpected medical expenses were prioritized.
  4.3. Accessibility Costs: Several participants reported that accessibility needs of their CMC necessitated expensive housing or larger vehicles. Others outlined the high cost of accessibility-related home renovations or vehicle adaptations, and difficulties in accessing funding subsidies for these expenses.

**Table 3.** Representative Quotations: Affected Domains of Parental Caregiver Experience.

Affected domains of parental caregiver experience	
Personal	
Identity	It was a complete fresh start, in every sense, from top to bottom—from how we eat, how we exercise, how we manage time, how we organize our life. Just everything, everything changed with [CMC]. (10) I didn’t know anything about motherhood, nothing about children. And I had to learn all that stuff, but I had to learn how to be a doctor, how to be a nurse, how to be a physiotherapist, an occupational therapist. (1) I mean I never thought in my life ever that I would be on social assistance. For me, that was always something for other people. Not for me. I’m ashamed to say I’m on social assistance. But I have no choice. (1)
Physical health	I’ve got the flexibility of an 80-year-old, I’m eating really badly: a bowl of cereal for breakfast, no lunch, and then whatever food is in front of my face at dinnertime, kind of as much as I can eat and then a couple hours up and I sleep. (3) Even getting a doctor’s appointment for one of us was really hard. We had to coordinate because one of us had to be with him all the time. Your health would suffer. There was a couple years where my wife would have wanted to have gone and it got punted down the road. (12)
Mental health	I feel like I have so much sadness that I don’t even know what to do with it or how to express it or how to live with it. (5) I have lots of panic attacks. I have lots of anxiety. I know I am depressed. They offered me a lot of medication, but the medication makes you sleepy and drowsy. I cannot afford to be sleepy or drowsy with [CMC]. (1) The fight is hard—we feel like we fight every single day, from the minute I wake up ‘til the minute I go to bed and fighting against an illness every day is exhausting as a caregiver. It feels like as a parent you’re just failing every single day, you always feel like you’re behind the game. And that’s really alienating, that’s why [families of CMC] burn out, because you just feel like you’re in it alone. (14)
Family	
Marriage	My wife and I, by and large, have been very supportive of each other. We tend to laugh about things, laugh together, make each other laugh. And we don’t blame, we try to solve; we’re very much a team and that makes going through difficult times a whole lot easier. (12) It’s taken a nosedive. Hundred percent. It’s a disaster. And that mainly comes from all of the added stress: the fact that we barely see each other, all of our conversations are centered around what’s going on with our kids in the hospital. Imagine having a roommate that is helping you take care of a sick kid. That’s kind of what it’s like. (3) I wonder if my husband and I will even have a relationship at the end of all this. You know once all this is over, I don’t know what we’re going to have left. (5)
Siblings	I worry about my relationship with my daughter. I worry that she’s going to resent me because I’ve spent so much time with [CMC] and I haven’t spent the same amount of time with her. (5) We’re seeing behaviour issues in the girls and issues affecting school. So, that’s been hard. I would say they’re not lacking in anything but they’re definitely struggling with us not being around all the time. (4) Her experience of being a sister of a sick kid, a kid with complications—she’s matured a lot in this time and has a different outlook on life. So it’s actually, indirectly, been good for her to have those experiences, unfortunately. (10)
Family quality of life	When [CMC] is doing well, everyone does well. And when he’s unwell we all get a little bit more on edge and a little bit more stressed, and everyone’s a little bit more tired. Things can deteriorate quickly. (6) With his illness, it’s just different, the dynamics. It affects what you’re able to do as a family. We used to do a lot of road trips, go do stuff on weekends. But since [CMC]’s been here those have been restricted, because of the medical needs and the extra baggage and the costs—all the factors associated. (12) Moving away from the city doesn’t make any sense for us right now. I do think his medical needs keep us where we are. Our home is perfect for the three of us right now, but we miss our own backyard and green space. It has dictated where we live and how we live, to be honest. (14)
Social	
Time limitations	It’s all I do, is the short answer. We don’t have a choice but to get out of bed in the morning and start feeds and get medication in and the same thing at nighttime, our bedtime is ruled by when he gets his medications. Everything I do is scheduled around his needs, it’s all consuming. (14) I don’t have any time for myself. I take about an hour, well it works out to about two hours a month. (5) I used to be very, very active in the community. I used to do a lot of volunteer work. I used to go out. I had lots of friends. But after having [CMC] it just—hospitals, appointments, doctors, sleepless nights, continuous work. That’s it. It’s just like—it’s just there’s no life at all. (1)
Isolated lived experience	What we deal with in a day, they don’t even deal with 1% of it in the whole of their life. So I don’t see why I’m sitting there with them. It’s like there is nothing in common anymore between me and any other person in the world. (1) The reality is people don’t understand if they haven’t been in a similar situation. Like “Oh, he’s home, he’s great now.” Well yeah he’s home, but it’s still giving him thirty-seven medicine doses every day, and if we give him a wrong dose he can die. (12) I just couldn’t talk about it and it was like the elephant in the room. Every time I saw people, people knew what was going on so it was just like we wouldn’t talk about it but it was there. (5)
Financial	
Employment	I used to work. My life was very good. I lost my job because [CMC] needs a very attention, like close watching. When you lose your job, everything you’re losing. (9) As much as I really need to go back to work because of my financial situation, I can’t do both. And if I have to pick one, I’m a dad first. (3) [My employer] accommodated my need to work from the hospital when I have to, but I have not been able to move on to other roles, even though I have very much outgrown the role that I’m in. I’ve been offered other opportunities, then when I explain what my accommodation needs are the offers are rescinded. (14)
Medical costs	Home and food and bills and there are a lot of costs associated with having a sick child—equipment, medical supplies, physiotherapies—not covered under Ontario Health Insurance Plan (OHIP). Those are the things that actually cost, on a month-to-month basis, quite a bit. It really means that we don’t have a nest egg, we have no plans for retirement, it’s hard to save anything. (14) [Government funding] doesn’t cover everything. And they tell you, “We’re not supposed to cover everything.” Then how are we supposed to cover it then? (1) There’s a decision that has to be made of “This is the cost of the marijuana, and this is what the quality of life is for our family, we’re just going to have to”—like it’s no question, we have to sacrifice in order to have that life for her. (4)
Accessibility costs	We’ve been working on a renovation; we’ve made a lift elevator in there and an accessible washroom. Unfortunately, because of my husband’s job and our wage earning we don’t qualify for any support or funding for any of the renos. We can’t move into a bungalow or smaller house for her wheelchair so this is just what we’re going to have to do and figure out how to budget over the next five years to pay it off. (4) Wheelchair van is in our future. Although how we’re going to afford it, I’m not entirely sure. (6)

Abbreviations: CMC, child(ren) with medical complexity.

### Determinants of Personal Resilience

**Figure 2.**
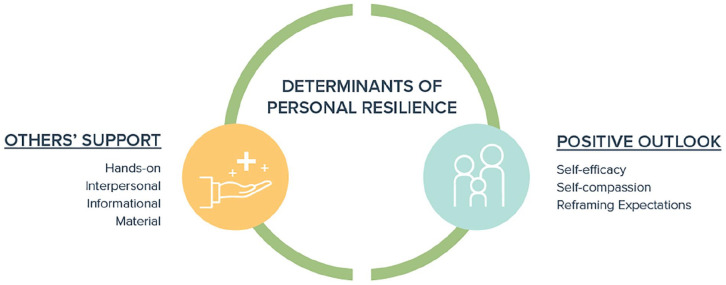
Determinants of personal resilience.

Despite substantial challenges experienced across these four domains, caregivers identified two core determinants of personal resilience: others’ support and a positive outlook. These are illustrated in Figure 2, with a selection of representative quotations in Table 4.

5. OTHERS’ SUPPORT
  5.1. Hands-on: Participants unanimously emphasized the support of hands-on caregiving by others. Some stressed the need for medically trained home nursing or personal support workers in addition to capable family and friends. In addition, several participants identified admission of their CMC to hospital as a relief from the total responsibility of caregiving, freeing time to dedicate to their own welfare or other relationships. Multiple participants identified the advantage of dependable programming or public schools designed for children with special needs.
  5.2. Interpersonal: Participants named a variety of sources of interpersonal and emotional support: their spouse, extended family, friends, religious community, home nurses, formal mental health care, and support groups for caregivers of CMC. Three participants identified validation provided by the Complex Care team as a particular source of support.
  5.3. Informational: Several participants named their medical teams as a valued expert with medical knowledge. The Complex Care team was uniquely highlighted for providing both medical information and resource navigation. Multiple other participants identified other parents of CMC as their most crucial resource for information on caregiving and advocacy.
  5.4. Material: Most participants mentioned the importance of material support given the significant impact of caregiving on family finances. Examples included extended family providing direct financial assistance, friends fundraising or providing food, workplaces allowing medical leave, and formal governmental and charity funding for medical supplies or personnel.
6. POSITIVE OUTLOOK
  6.1. Self-efficacy: Several caregivers attributed improved coping to a belief in themselves and their caregiving ability. Two participants described feeling chosen or born to fulfill this caregiving role. Some outlined personal characteristics which made them well-suited to caregiving; others explained that acquiring expertise through lived experience of caregiving for their CMC fostered resilience and empowered them to collaborate with the health care team.
  6.2. Self-compassion: Some caregivers expressed self-awareness and compassion for their physical and emotional caregiving limitations, encouraged by self-reflection, belief in God, or a supportive medical team. Two participants emphasized pacing oneself to avoid burnout. Several others offered advice to encourage other caregivers to ask for support.
  6.3. Reframing Expectations: Caregivers described a positive reframing of their expectations of their CMC, family unit, career, and social commitments; one participant reported how caregiving refocused their family on the “big picture.” Many participants described acceptance of their CMC by overcoming their own preconceptions and not heeding others’ expectations of ‘normal.’ A few participants emphasized the value to parents, siblings, and the CMC of adapting plans to facilitate full family participation. Participants who perceived good quality of life for their CMC focused on their child’s happy disposition and strong interpersonal relationships.

**Table 4.** Representative Quotations: Determinants of Personal Resilience.

Determinants of personal resilience	
Others’ support	
Hands-on	Since we’ve received that overnight [nursing] assistance, that has helped immensely. The nights when I work the next day I don’t have to be sleeping on the couch beside her room, and really you’re awake most of the time. That’s been an enormous help, it’s become a little bit easier. (13) People usually don’t feel good about coming to the hospital. But in my case, I would always prefer to come to the hospital, because I know he will get the good care in here, and I will get my time too. I feel like more relaxed here, rather than home, because I have to take care of him at home completely. (2) He was fortunate to get into a school that was, that is for special needs children, so again they understand his needs and they focus their programming around special needs and addressing his needs. (6)
Interpersonal	People praying for us, people praying with us, us praying, praying together, that’s been a big part of how we’ve been able to cope. Our church family supporting us is a big piece. (12) [Caregiver Facebook groups are] like a big family, even though we don’t even see each other. We don’t know how we look like, but we know each other’s problems. It’s just more like some emotional support. (1)
Informational	As long as I have the Complex Care team, then I know that I can get through each day, because I’ve got that support. I just know that when I feel like I don’t understand what’s going on, I don’t know what to do, I know that someone there will be able to give me direction. (7) There’s a huge disconnect when there’s a patient who has multiple issues and there’s multiple departments that need to be dealt with. Complex Care listened to us and actually worked with us to get the right people at the right time. (10) Most of the things I learned in my life with my son is through other mothers, not through professionals. Very, very little I learned from professionals. Everything is from other mothers. (1)
Material	The first year, [friends] raised $50,000 for our family. That allowed us to get a nurse and then all the visits and travels [to the hospital from home] and food down here, all those little things add up. (4) The last year that we’ve gotten that [government program], that has really changed things where we’ve had the ability to have overnight help that we did not have to pay for. We could afford maybe one, two nights tops, where now we have five and it’s freed up money to increase the personal support worker (PSW) hours. (13) Thank God for the [charities] because they allowed me to have a new accessible van. That made a huge difference, positively, in my life. (1)
Positive outlook	
Self-efficacy	Before, when [CMC] was born, I was saying “Why me? Why me? Why does it have to be me?” But after all, I said “Maybe I’m a chosen person. Maybe that’s why she’s in my life.” We have to focus to all our kids. We have to hold a candle in our hand and show them the way. (9) Medically we know a lot, not because of our schooling or our education or anything like that, but because of what we’ve been through. (15) I would hear a word and I would Google it, I would read up what it meant and some of the ways to resolve it, and when we’d discuss it again I’d have a little bit more knowledge. It got to a point where I would be able to tell the doctors, “Hey no, we’ve already done that if you look back, why haven’t we done this?”. It allowed us to kind of navigate her care. Families are needed to bridge the gap, to complete the picture. (10)
Self-compassion	In the times in his life when [CMC] almost didn’t make it, then I’d say we aren’t able to cope. I mean, I don’t think anybody’s able to cope with that, but you just do it anyways. (15) I think an acceptance. We can treat [her seizures] the best we can but this is going to be our reality. And then just the team here coming around and going “Okay [Mom], you’re not going to catch every single seizure. You can’t physically watch her every second.” (4) Reaching out to as many services as you possibly can is my biggest advice. Because in the first few years we really didn’t take advantage of that, and it was extremely difficult. Reach out for as much help as possible; don’t try to shoulder the burden all by yourself. (13)
Reframing expectations	He, in and of himself, is very happy. He is by far the happiest of [his siblings]. So having robust health is not necessarily an indicator of happiness. And he is happy as a clam. (8) It hasn’t really affected my finances, more just my time. So if you want to say ‘time is money’ then sure. But [CMC]’s also more valuable than money, so it’s time well spent. (10) It was just like zooming out of my family situation and going “Whoa, I can see the bigger picture here. And it’s alright, this is life. It just looks different than I ever thought.” (4) You’re sitting there on the beach, and you’ve got your oxygen, and you’ve got your suction equipment, and you’ve got your feed equipment. And here’s my husband and I on a big blanket, and we’re deep suctioning on the beach. And it’s a little crazy, but we do it all for our kids. (8)

Abbreviation: CMC, child(ren) with medical complexity.

## Discussion

The field of Complex Care was developed with a strong focus on providing medical service and care coordination to improve care delivery for CMC.^4,23^ It has more recently been suggested that to optimize outcomes for CMC, one must support both the child and the family, as strengthening resilience in caregivers allows them to better meet their child’s needs; to care for the caregiver is to care for the child.^24-28^ This study explores factors influencing wellness of the caregiver and family of CMC, thereby highlighting opportunities for health care provider intervention.

In our study, parental caregivers highlight adverse effects on their sense of self, social attachments, and finances as a consequence of caring for a CMC. Consistent with previous literature in medically fragile children, participants in our study identify incongruence in the dual identities of parent and medical carer.^29-32^ This conflict contributes to caregivers’ negative mental health outcomes and feelings of social isolation.^33,34^ Beyond the personal impact, themes of marital discord and limited available time for other family members raised by participants have also been previously described across pediatric patient populations.^31-33,35-37^ One study suggests that siblings of children with disabilities have some degree of unintentional neglect;^38^ this may be more marked in siblings of CMC given greater caregiving need. In addition, complexity of planning activities involving CMC is a barrier to valued social and family time.^39^ Health care providers of CMC must support caregivers in navigating their multiple individudal and relational identities: as medical carer, parent to the CMC and their siblings, spouse, and friend. Health care providers can help caregivers mitigate time demands and seek balance by adopting a mindful approach when suggesting medication and nutrition schedules (eg, streamlining medication administration times; taking into account the family’s typical schedule when recommending timing of enteral feeds), and by seeking alternative caregivers for CMC through respite services.

Together with the adversity associated with caregiving, participants described how resilience in caregiving can be realized with appropriate supports and a positive cognitive framework. The benefits to primary caregivers of instrumental support through shared carer responsibilities^32,40,41^ and access to respite services^41-44^ have been emphasized in the pediatric literature. Participants in this study uniquely identify hospitalization of their CMC as a form of personal respite; while seemingly paradoxical, this suggests the high level of distress experienced by caregivers of CMC at home. Aligned with previous research, other necessary forms of support identified include emotional comfort,^8,31,32,36,41,45,46^ financial aid,^32,41,47,48^ and provision of caregiving-related information from peers and professionals.^40,45,47,48^ In one striking example, a recent study found that receiving up-to-date written care plans was associated with significantly fewer depressed and anxious days in caregivers of CMC.^49^ To promote caregiver resilience, health care providers of CMC must identify and make appropriate referrals to existing programs for hands-on and financial support, and advocate for increased access and funding for these invaluable resources. Clinicians should also provide families with informational support, such as a written document outlining the CMC’s health conditions and care needs,^48,50^ and seek opportunities for interpersonal connection for caregivers.

Another key contributor to resilience which emerged from this study is personal growth, stemming from a conceptual framework emphasizing the caregivers’ own strength and that of their families. This echoes previous studies on caregiving for a child with disability,^31,36,46,51-53^ as well as definitions of caregiver resilience posited by other studies and reviews.^54,55^ Caregiving promotes empathy and fortitude, and focuses families on that which gives their lives meaning.^34^ Resilient caregivers in our study recognized factors that increased quality of life for their CMC and the favorable impact their child has on their family unit. Health care providers should use a strengths-based approach to promote acceptance and a positive concept of the child, self, and family. Finally, resilient participants recognized their limitations and exercised self-compassion. Drawing on their roles as both information provider and interpersonal support, health care providers should initiate explicit discussion with parents of CMC about caregiving expectations and caregiver burnout.

This article supports the evolution of the field of Complex Care beyond the core components of medical technology and comprehensive care planning for the pediatric patient, toward a recognition of the family as a holistic unit requiring intervention and advocacy through medical and social supports.

### Limitations

Self-selection in the caregivers who participated may have influenced the data collected given the study’s sample size. For instance, most participants were married and employed at the time of the interview—features not reflective of all caregivers for CMC. In addition, non–English speaking caregivers were excluded who likely face additional barriers to resource navigation and health care access. Caregivers of CMC at end-of-life were also excluded; while qualitative research has been conducted by members of this research team on caregiver perspectives on advanced care planning,^56^ future studies should address the caregiving experience and needs of this specific population. Thirteen of fifteen participants lived in urban settings, therefore rural caregiving experiences may not be captured. This study is limited to the Canadian context and does not reflect international health care settings with different services and payment models. Finally, though common themes emerged among the represented range of CMC diagnoses, impacts of caregiving for CMC with specific diagnoses or treatments may have been missed.

## Conclusion

Children with medical complexity have a lifelong need for caregiving resulting in far-reaching implications for their parental caregivers. Participants in this qualitative study relay a diversity of experiences related to caregiving for CMC. Negative effects of caregiving are felt across personal, family, social, and financial domains; yet, resilience is fostered through others’ support and maintaining a positive outlook. This study helps lay the foundation for subsequent research examining the unique and multilayered needs and strengths of caregivers for this vulnerable population.

## Author Contributions

JT: Contributed to conception and design; contributed to acquisition, analysis, and interpretation; drafted the manuscript; gave final approval; agrees to be accountable for all aspects of work ensuring integrity and accuracy.

CM: Contributed to analysis and interpretation; contributed to drafting the manuscript; critically revised the manuscript; gave final approval; agrees to be accountable for all aspects of work ensuring integrity and accuracy.

KE: Contributed to acquisition, analysis, and interpretation; contributed to drafting the manuscript; critically revised the manuscript; gave final approval; agrees to be accountable for all aspects of work ensuring integrity and accuracy.

NW: Contributed to conception and design; contributed to acquisition, analysis, and interpretation; critically revised the manuscript; gave final approval; agrees to be accountable for all aspects of work ensuring integrity and accuracy.

DA: Contributed to analysis and interpretation; critically revised the manuscript; gave final approval; agrees to be accountable for all aspects of work ensuring integrity and accuracy.

EC: Contributed to conception and design; critically revised the manuscript; gave final approval; agrees to be accountable for all aspects of work ensuring integrity and accuracy.

JO: Contributed to conception and design; contributed to analysis and interpretation; critically revised the manuscript; gave final approval; agrees to be accountable for all aspects of work ensuring integrity and accuracy.
